# Return to play after hip arthroscopy among tennis players: outcomes with minimum five-year follow-up

**DOI:** 10.1186/s12891-020-03424-w

**Published:** 2020-06-23

**Authors:** David R. Maldonado, Mitchell J. Yelton, Philip J. Rosinsky, Jacob Shapira, Mitchell B. Meghpara, Ajay C. Lall, Benjamin G. Domb

**Affiliations:** 1 American Hip Institute Research Foundation, Des Plaines, IL 60018 USA; 2 AMITA Health St. Alexius Medical Center, Hoffman Estates, IL 60169 USA; 3 American Hip Institute, 999 E Touhy Ave, Suite 450, Des Plaines, IL 60018 USA

**Keywords:** Hip arthroscopy, Return to sport, Patient-reported outcomes

## Abstract

**Background:**

Playing tennis is associated with various movements that can lead to labral injuries and may require arthroscopic surgery. While hip arthroscopies have demonstrated good outcomes in athletes, there is limited literature reporting patient reported outcomes (PROs) and return to sport (RTS) in competitive or recreational tennis players after arthroscopic hip surgery. Therefore, the purpose of the present study was to (1) report minimum five-year PROs and RTS in tennis players who underwent arthroscopic hip surgery and (2) compare outcomes between recreational and competitive tennis players.

**Methods:**

Data for patients who underwent hip arthroscopy surgery in the setting of femoroacetabular impingement and labral tears between March 2009 and January 2014 and who played tennis within one-year of surgery were retrospectively reviewed. Patients with preoperative and minimum five-year postoperative scores for the following PROs were included: modified Harris Hip Score (mHHS), Non-Arthritic Hip Score (NAHS), Hip Outcome Score-Sport Specific Subscale (HOS-SSS), and visual analog scale (VAS) for pain. Patient Acceptable Symptomatic State (PASS) and Minimal Clinically Important Difference (MCID) for mHHS and HOS-SSS were calculated.

**Results:**

Of 28 patients, 31 hips met all inclusion and exclusion criteria of which 28 (90.3%) had minimum 5-year follow-up (mean: 72.8 ± 13.9 months). There were 3 professional, 3 collegiate, 2 high school, 2 organized amateur, and 18 recreational level tennis players. All PROs significantly improved at latest follow-up: mHHS from 67.0 to 86.7 (*P* <  0.001), NAHS from 65.9 to 87.2 (*P* <  0.001), HOS-SSS from 50.0 to 77.9 (*P* = 0.009), and VAS from 5.4 to 1.8 (*P* <  0.001). There was a 75.0% RTS rate. Additionally, 66.7% of patients achieved MCID and 83.3% achieved PASS for mHHS, and 63.6% achieved MCID and 58.3% achieved PASS for HOS-SSS.

**Conclusion:**

Regardless of the level of participation, tennis players who underwent arthroscopic hip surgery reported statistically significant PRO improvements. A favorable rate of RTS was also achieved by players with a continued interest in playing. The data here may be useful in counseling tennis players of various levels who are considering arthroscopic treatment of a hip injury.

**Level of evidence:**

IV

## Background

The popularity of tennis has increased in recent years, becoming one of the most popular sports across the world, with over 75 million participants worldwide [1]. According to the Tennis Industry Association (TIA), almost 18 million Americans play tennis and another 14 million show interest in the sport. Playing tennis is associated with rotational stresses, cutting movements, and repetitive loading, which can lead to labral injuries [2]. Injuries of the hip joint account for 6% of sport injuries [2]. For tennis specifically, hip joint injuries may account for up to 27% of the sport’s injuries [2]. Surgical treatments for hip injuries have been revolutionized with hip arthroscopy, which have demonstrated good outcomes in athletes [3–7]. Byrd and Jones reported successful long-term outcomes after arthroscopic management of femoroacetabular impingement (FAI) in athletes [8]. Weber et al. examined return to play in professional and recreational athletes of a variety of sports following hip arthroscopy for treatment of FAI. They reported that recreational and professional athletes returned to their sport at similar rates and had comparable satisfaction and patient reported outcomes (PROs) [9]. In a systematic review, 87% of athletes with symptomatic FAI returned to their respective sport after hip arthroscopy, and 82% returned to the same level of play prior to the onset of their symptoms [10]. To our knowledge, there is limited data in the literature reporting PROs and return to play in competitive or recreational tennis players after arthroscopic hip surgery. The purpose of the present study was to (1) report minimum five-year PROs and rate of return to sport (RTS) in tennis players who underwent arthroscopic hip surgery and to (2) compare outcomes between recreational and competitive tennis players. Our hypothesis was that both competitive and recreational tennis players would have improved PROs after hip arthroscopy and would return to tennis at similar rates.

## Methods

### Patient selection criteria

Data were prospectively collected and retrospectively reviewed for all patients who underwent primary hip arthroscopy in the setting of FAI or a labral tear between March 2009 and January 2014. Patients who met any of the following criteria were excluded from this study: patients over 60 years old, Tönnis osteoarthritis Grade > 1, Workers’ Compensation claims, previous ipsilateral hip surgeries, or previous hip conditions such as Perthes, avascular necrosis, slipped capital femoral epiphysis, femoral head or acetabulum fractures. Patients in this study played tennis at the professional, collegiate, high school, organized amateur, or recreational level within one-year of surgery and intended to return to tennis following surgery. Patients who had preoperative and minimum five-year follow-up on the following outcome measures were included in this analysis: modified Harris Hip Score (mHHS), Non-Arthritic Hip Score (NAHS), Hip Outcome Score-Sport Specific Subscale (HOS-SSS), and visual analog scale (VAS) for pain.

All patients participated in the American Hip Institute Hip Preservation Registry. While the present study represents a unique analysis, data on some patients in this study has been reported in other studies [11, 12]. All data collection received Institutional Review Board approval.

### Indications for surgery

All surgical candidates were assessed with a detailed medical history, physical examination, and radiographic analysis. Patients were evaluated for FAI, acetabular version, dysplasia, and Tönnis grade for osteoarthritis using the supine, anteroposterior pelvis, false-profile, and Dunn-view X-ray views. In addition, magnetic resonance arthrography (MRA) was used to assess labral tears and cartilage damage. If patients had pain interfering with the activities of daily living for at least 3 months and failed to improve with conservative measures (rest, non-steroidal anti-inflammatory drugs (NSAIDs), cortisone injections, and physical therapy), they were recommended for surgery.

### Surgical technique

All surgeries were performed by a single surgeon (B.G.D.) with the patients placed in the supine position on a traction table with a well-padded perineal post [13, 14]. The joint was accessed through the standard anterolateral and mid-anterior accessory portals. A capsulotomy was performed with a beaver blade. The intra-articular space was then evaluated using a diagnostic arthroscopy. The Seldes [15], acetabular labral articular disruption (ALAD) [16], and Domb and Villar Classification Systems [17] were used to assess the labrum, intra-articular cartilage, and ligamentum teres, respectively. An acetabuloplasty was performed to address pincer-type FAI and a femoroplasty was performed to address cam-type FAI [18–20]. Ligamentum teres (LT) tears were debrided [21]; full-thickness chondral defects were addressed with microfracture [22]; and iliopsoas impingement lesions or painful internal snapping were treated with iliopsoas fractional lengthening [23, 24]. Labral tears were treated with repair, debridement, resection, or reconstruction depending on the integrity of the labrum [25]. The decision to release, repair, or plicate the capsule was dictated by the patient’s Beightons score and acetabular coverage [26, 27].

### Rehabilitation

Using a fitted X-Act ROM brace (DJO Global Vista, CA) and crutches, patients were instructed to use toe-touch weight bearing for 2 weeks. To restore strength and range of motion, physical therapy was initiated 1 day after surgery. Rehabilitation plans were adjusted for patients who underwent labral reconstruction, gluteus medius repair, or microfracture [28].

### Patient-reported outcomes

Outcomes were assessed preoperatively and postoperatively at 3 months, 1-year, and annually thereafter using the following PROs: mHHS [29], NAHS [30], HOS-SSS [31], and VAS for pain [32]. Postoperative values for the International Hip Outcome Tool (iHOT-12) [33], the physical and mental components of the Veterans RAND 12-Item Health Survey (VR-12P and VR-12 M, respectively), and the physical and mental components of the Short Form 12 (SF-12P and SF-12 M, respectively) were also collected. Preoperative values for these scores were unavailable as they were not routinely collected for patients in this study period. Additionally, for mHHS and HOS-SSS, the number of patients achieving minimal clinically important difference (MCID) (+ 8 and + 6, respectively) and patient acceptable symptomatic state (PASS) (≥ 74 and ≥ 75, respectively) were calculated [34]. VAS was measured on a scale from 0 (no pain) to 10 (worst possible pain). Patient satisfaction was rated out of 10, with 10 signifying extreme satisfaction. Complications, secondary arthroscopies, and conversion to total hip arthroplasty (THA) were also reported.

### Sub-analysis

A sub-analysis was performed to compare those who returned to tennis following surgery to those who were not able to return to play following surgery. An additional sun-analysis was performed to compare hips with severe cartilage damage (Acetabular Outerbridge grade > 2 and/or Femoral head Outerbridge grade > 2) to those without severe cartilage damage. These analyses compared age, body-mass index (BMI), rate of RTS, and pre- and postoperative PROs.

### Statistical analysis

All statistical analyses were completed using Microsoft Excel with the Real Statistics Add-In (Microsoft Corporation; Redmond, WA). Normality and equality of variances were assessed by the Shapiro-Wilk Test and F-test, respectively. The two-tailed student’s T-test assessed continuous parametric data. The Wilcoxon signed-Rank test was used as the non-parametric equivalent. Spearman’s rank correlation coefficient was used to assess the correlation between physical exam findings and RTS ability. Categorical data was evaluated using the Chi-Square and Fisher’s exact tests. A *P*-value of < 0.05 was considered statistically significant.

## Results

### Patient demographics

There were 28 patients (31 hips) that met the inclusion and exclusion criteria. Of these, 25 (89.3%) patients (28 hips) had minimum five-year follow-up (mean: 72.8 ± 13.9 months). There were 3 professional, 3 collegiate, 2 high school, 2 organized amateur, and 18 recreational level tennis players. This patient cohort consisted of 14 (50.0%) males and 14 (50.0%) females. The mean age at surgery was 41.4 ± 12.7 years and the mean BMI was 25.1 ± 3.9 kg/m^2^. The demographics are summarized in Table 1.

**Table 1:** Demographics

	Value
Patients and hips included in study	25 patients, 28 hips
Left	10 (35.7%)
Right	18 (64.3%)
Gender	
Male	14 (50.0%)
Female	14 (50.0%)
Age at surgery (years, mean, SD, range)	41.4 ± 12.7 (14.5–70.0)
BMI (mean, SD, range)	25.1 ± 3.9 (18.3–34.2)
Follow-up time (months, mean, SD, range)	66.8 ± 20.2 (60.0–114.2)
Follow-up percentage	90.32%
Flexion° (mean, SD, range)	116.3 ± 14.8 (85–140)
Internal Rotation° (mean, SD, range)	20.0 ± 14.5 (5–55)
External Rotation° (mean, SD, range)	42.5 ± 13.6 (15–70)

*BMI* Body mass index, *SD* Standard deviation

### Intraoperative findings

Intraoperative findings from the diagnostic arthroscopy are summarized in Table 2. All patients had a labral tear. There were 12 (42.9%) Seldes Type 1, 4 (14.3%) Seldes type II, and 12 (42.9%) Seldes type I & II labral tears. Cartilage integrity was assessed using the ALAD and Outerbridge classification systems. Twenty-four (85.7%) hips were assigned an ALAD grade ≥ 2, 25 (89.3%) hips were assigned an acetabular Outerbridge grade ≥ 2, and 12 (42.9%) hips were assigned a femoral head Outerbridge grade ≥ 2. Fourteen (50%) hips presented with LT tears.

**Table 2:** Intraoperative findings

	n (%)
Seldes Tear Type	
0	0
1	12 (42.9%)
2	4 (14.3%)
1 & 2	12 (42.9%)
ALAD	
0	1 (3.6%)
1	3 (10.7%)
2	10 (35.7%)
3	10 (35.7%)
4	4 (14.3%)
Outerbridge (Acetabular)	
0	0 (0.0%)
1	3 (10.7%)
2	10 (35.7%)
3	5 (17.9%)
4	10 (35.7%)
Outerbridge (Femoral Head)	
0	16 (57.1%)
1	0 (0.0%)
2	6 (21.4%)
3	3 (10.7%)
4	3 (10.7%)
LT Percentile Class (Domb)	
0–0%	14 (50.0%)
1–0% < 50%	7 (25.0%)
2–50% < 100%	7 (25.0%)
3–100%	0 (0.0%)
LT Villar Class^a^	
0 - No tear	14 (53.8%)
1 - Complete Rupture	0 (0%)
2 - Partial Tear	9 (34.6%)
3 - Degenerate Tear	3 (11.5%)

*ALAD* Acetabular labral articular disruption, *LT* Ligamentum teres

^a^Only data on 26 of 28 patients

### Arthroscopic procedures

The intraoperative procedures are summarized in Table 3. The majority (64.3%) of labral tears were repaired. The capsule was repaired or plicated in 12 (42.9%) hips and released in 16 (57.1%) hips. Twenty-five (89.3%) hips underwent a femoroplasty and 21 (75.0%) hips underwent an acetabuloplasty. Of the 14 LT tears, 12 (85.7%) hips were treated via debridement. Additionally, 10 (35.7%) hips underwent an iliopsoas fractional lengthening.

**Table 3:** Procedures

	n (%)
Labral treatment	
Repair	18 (64.3%)
Debridement	8 (28.6%)
Resection	1 (3.6%)
Reconstruction	1 (3.6%)
Capsular Treatment	
Repair/Plication	12 (42.9%)
Release	16 (57.1%)
Femoroplasty	25 (89.3%)
Acetabuloplasty	21 (75.0%)
Iliopsoas fractional lengthening	10 (35.7%)
Ligamentum teres debridement	12 (42.9%)
Removal of loose body	7 (25.0%)
Synovectomy	8 (28.6%)
Trochanteric bursectomy	6 (21.4%)
Gluteus medius/minimus repair	5 (17.9%)
Acetabular microfracture	6 (21.4%)
Femoral head microfracture	2 (7.1%)
Acetabular chondroplasty	6 (21.4%)
Femoral head chondroplasty	4 (14.3%)

### Outcomes at latest follow-up

Preoperative and minimum five-year PROs, VAS, and patient satisfaction are detailed in Table 4. All mean scores improved significantly at latest follow-up: mHHS improved from 67.0 to 86.7 (*P* <  0.001), NAHS improved from 65.9 to 87.2 (*P* <  0.001), HOS-SSS improved from 50.0 to 77.9 (*P* = 0.009), and VAS improved from 5.4 to 1.8 (*P* <  0.001). For mHHS, 66.7% of patients achieved MCID and 83.3% achieved PASS. For HOS-SSS, 63.6% achieved MCID and 58.3% achieved PASS. Mean patient satisfaction with surgery was 8/10. There was a 75.0% RTS rate in this tennis population, with 15 (71.4%) playing at the same or a higher level postoperatively. Although not statistically significant, patients who played tennis at a competitive level (professional, college, high school, or organized amateur) experienced greater improvements in outcome scores compared to patients who played tennis recreationally (Table 5). The nature of our data suggest that competitive athletes may achieve higher PROs at minimum 5-year follow-up compared to the recreational group, particularly for HOS-SSS and VAS (Table 5). The mean age of the two groups were not statistically different at 34.9 years in the competitive group and 44.0 years in the recreational group (*P* = 0.099).

**Table 4:** PROs

	Preoperative	Minimum Five-Year Follow-Up	***P***-Value
mHHS (mean, SD)	67.0 ± 17.2	86.7 ± 16.4	**< 0.001**
NAHS (mean, SD)	65.9 ± 17.3	87.2 ± 17.4	**< 0.001**
HOS-SSS (mean, SD)	50.0 ± 25.0	77.9 ± 25.2	**0.009**
IHOT (mean, SD)		80.7 ± 26.6	
SF-12 Mental (mean, SD)		58.8 ± 3.0	
SF-12 Physical (mean, SD)		51.9 ± 8.3	
VR-12 Mental (mean, SD)		63.4 ± 3.5	
VR-12 Physical (mean, SD)		52.9 ± 8.0	
VAS (mean, SD)	5.4 ± 2.3	1.8 ± 2.5	**< 0.001**
Patient Satisfaction (mean, SD)		8.0 ± 3.2	

Bold text: statistically significant

*PROs* Patient reported outcomes, *mHHS* Modified Harris Hip Score, *NAHS* Non-Arthritic Hip Score, *HOS-SSS* Hip Outcome Score-Sport Specific Subscale, *VAS* Visual analog scale, *iHOT-12* International Hip Outcome Tool, *VR-12P and VR-12 M* The physical and mental components of the Veterans RAND 12-Item Health Survey, respectively, *SF-12P and SF-12 M, respectively* The physical and mental components of the Short Form 12, respectively

**Table 5:** Comparison of PROs between high level and recreational tennis players

	Competitive (***n*** = 12)	Recreational (***n*** = 18)	***P***-value
mHHS (mean, SD)			
Pre	69.7 ± 12.6	65.9 ± 18.9	0.607
Latest	95.6 ± 8.3	83.0 ± 17.7	0.075
Pre-Post P-Value	**0.016**	**0.009**	
Δ	25.8 ± 16.4	17.5 ± 25.2	0.433
NAHS (mean, SD)			
Pre	72.0 ± 16.2	63.5 ± 17.5	0.249
Latest	94.6 ± 10.4	84.2 ± 19.0	0.130
Pre-Post P-Value	**0.047**	**0.002**	
Δ	20.4 ± 18.0	20.3 ± 24.2	0.991
HOS-SSS (mean, SD)			
Pre	54.9 ± 25.0	48.0 ± 25.4	0.521
Latest	91.7 ± 22.0	71.9 ± 24.7	**0.027**
Pre-Post P-Value	0.063	0.058	
Δ	34.9 ± 36.4	20.8 ± 33.7	0.381
VAS (mean, SD)			
Pre	5.4 ± 1.8	5.4 ± 2.5	0.98
Latest	0.4 ± 0.8	2.4 ± 2.8	**0.041**
Pre-Post P-Value	**0.016**	**< 0.001**	
Δ	−4.8 ± 2.0	−3.2 ± 2.5	0.146
IHOT (mean, SD)	91.4 ± 18.5	76.0 ± 28.6	0.062
SF-12 Mental (mean, SD)	59.0 ± 3.2	58.8 ± 3.0	0.535
SF-12 Physical (mean, SD)	55.5 ± 2.4	50.4 ± 9.5	0.058
VR-12 Mental (mean, SD)	64.4 ± 2.7	63.0 ± 3.8	0.535
VR-12 Physical (mean, SD)	56.3 ± 2.1	51.4 ± 9.1	0.058
Patient Satisfaction (mean, SD)	8.4 ± 2.9	7.8 ± 3.4	0.757

Bold text: statistically significant

*PROs* Patient reported outcomes, *Δ* Delta, *mHHS* Modified Harris Hip Score, *NAHS* Non-Arthritic Hip Score, *HOS-SSS* Hip Outcome Score-Sport Specific Subscale, *VAS* Visual analog scale, *iHOT-12* International Hip Outcome Tool, *VR-12P and VR-12 M* The physical and mental components of the Veterans RAND 12-Item Health Survey, respectively, *SF-12P and SF-12 M, respectively* The physical and mental components of the Short Form 12, respectively

### Sub-analysis on return to sport

**Fig. 1:**
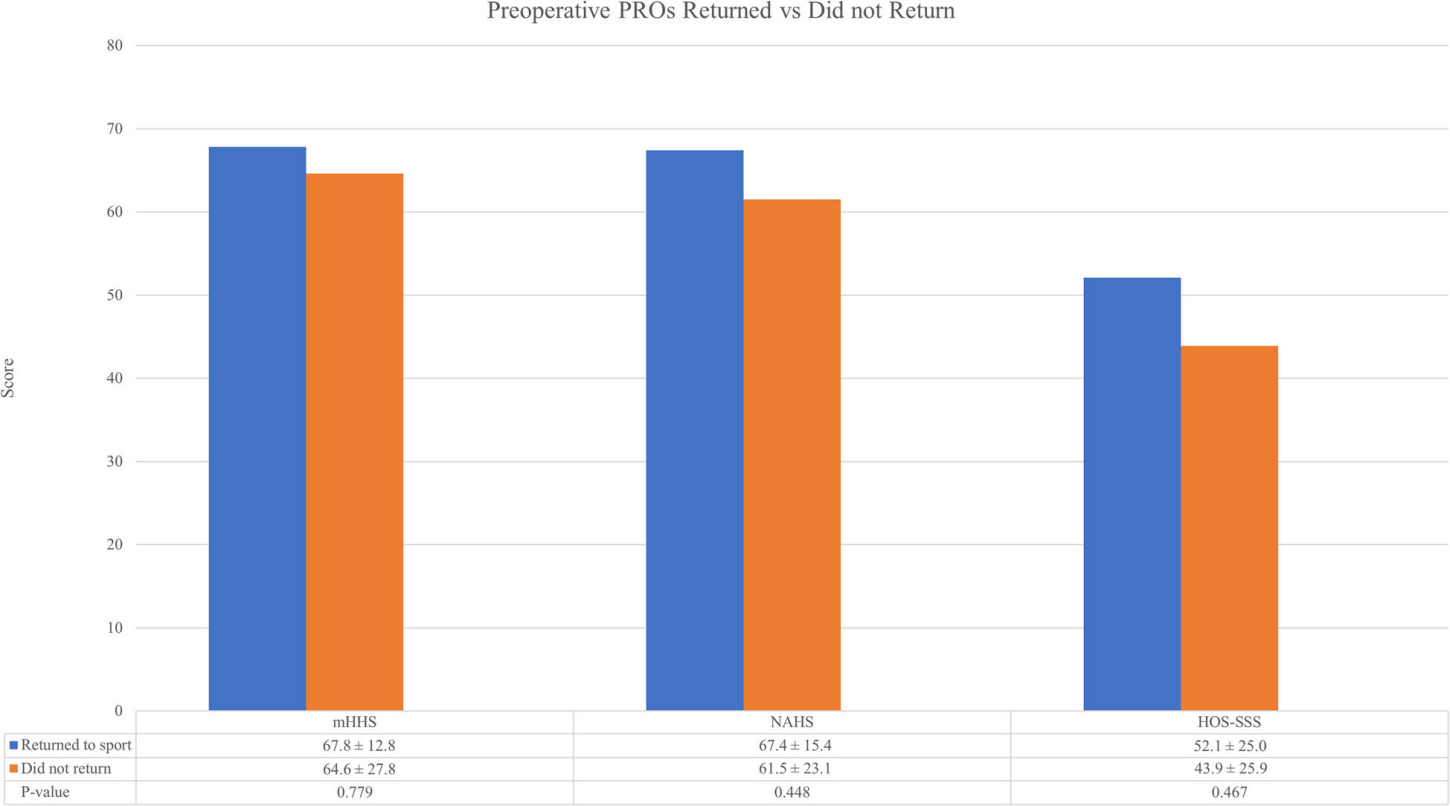
(PROs), patient reported outcomes, (mHHS), modified Harris Hip Score, (NAHS), Non-Arthritic Hip Score, (HOS-SSS), Hip Outcome Score-Sport Specific Subscale

**Fig. 2:**
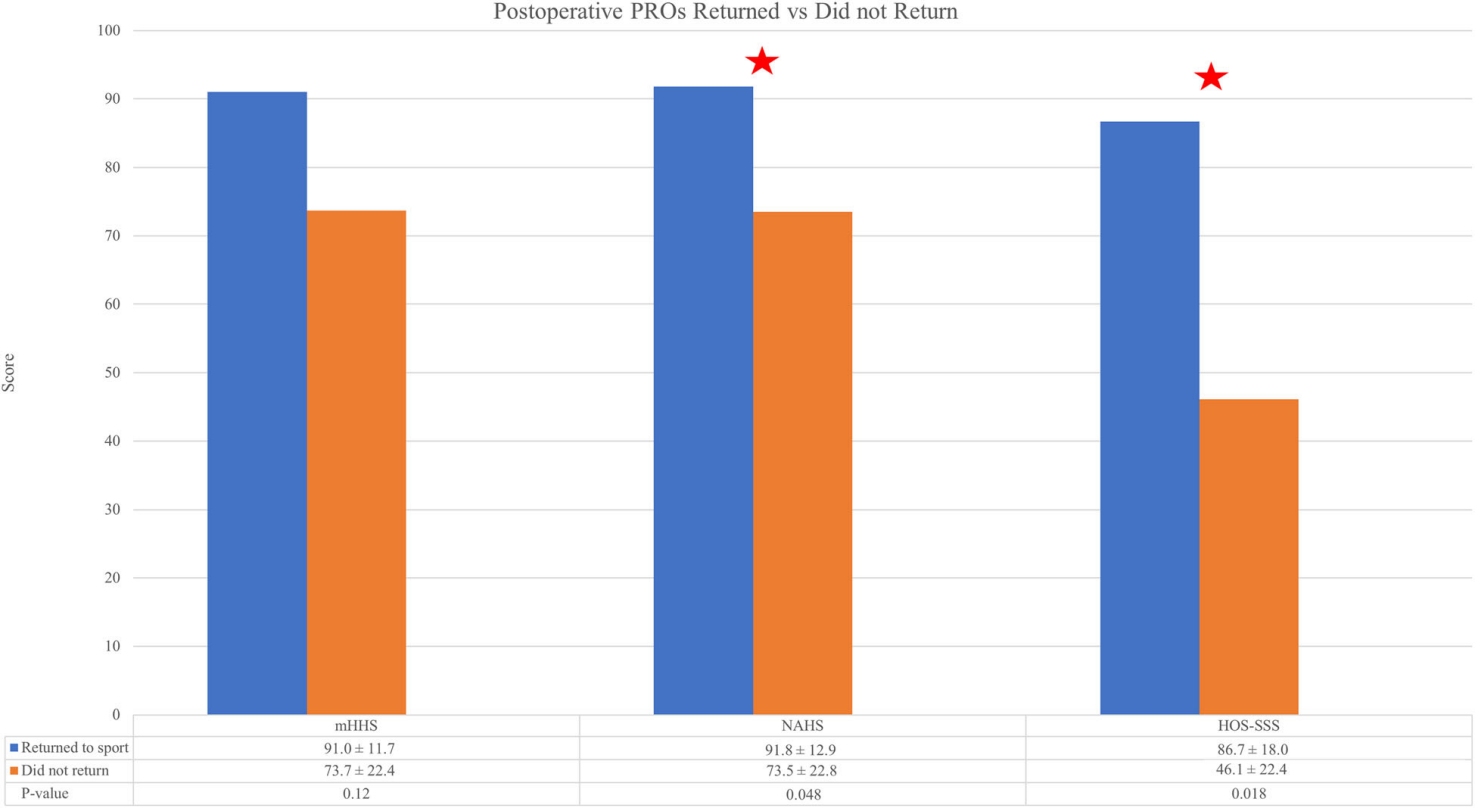
(PROs), patient reported outcomes, (mHHS), modified Harris Hip Score, (NAHS), Non-Arthritic Hip Score, (HOS-SSS), Hip Outcome Score-Sport Specific Subscale

Both competitive and recreational tennis players returned to play at similar rates (*P* = 0.233). There seemed to be a pattern in patients who returned to tennis having higher preoperative mHHS, NAHS, and HOS-SSS scores; however, there was no significant differences (*P* > 0.05) between the RTS and did not RTS groups (Fig. 1). Although only statistically significant for NAHS and HOS-SSS (*P* = 0.048 and 0.018, respectively), there was a similar trend suggesting patients who were able to return to tennis at latest follow-up had superior PROs at minimum 5-year follow-up compared to patients who were not able to return to tennis (Fig. 2). In addition, there was no significant differences (*P* > 0.05) in age or BMI between the RTS and did not RTS groups. With regard to physical examination findings, the relationship indicated a negative correlation between flexion, internal rotation, external rotation and RTS ability, however these were not statistically significant (*P* > 0.05).

### Sub-analysis on cartilage damage

**Fig. 3:**
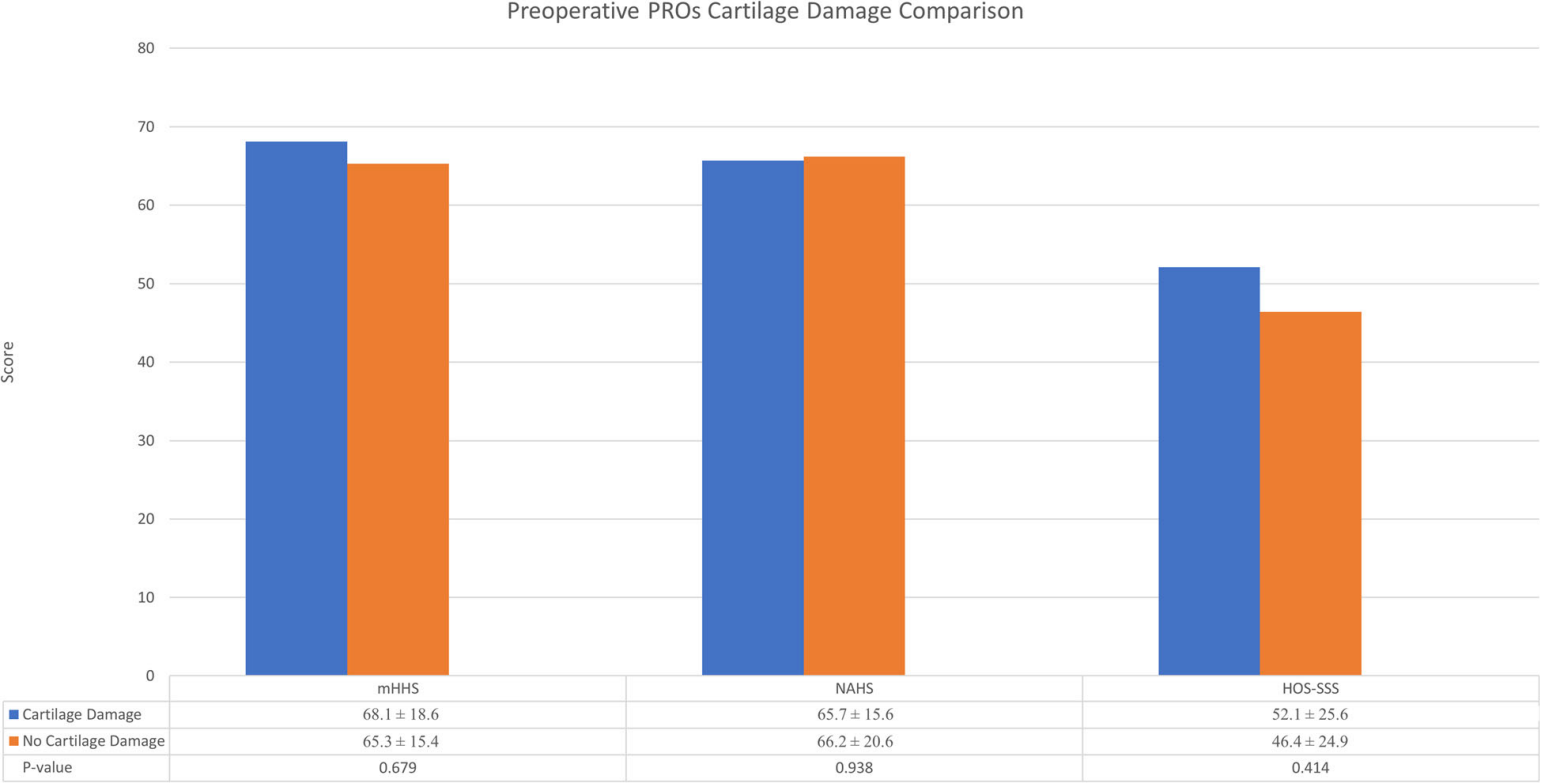
(PROs), patient reported outcomes, (mHHS), modified Harris Hip Score, (NAHS), Non-Arthritic Hip Score, (HOS-SSS), Hip Outcome Score-Sport Specific Subscale

**Fig. 4:**
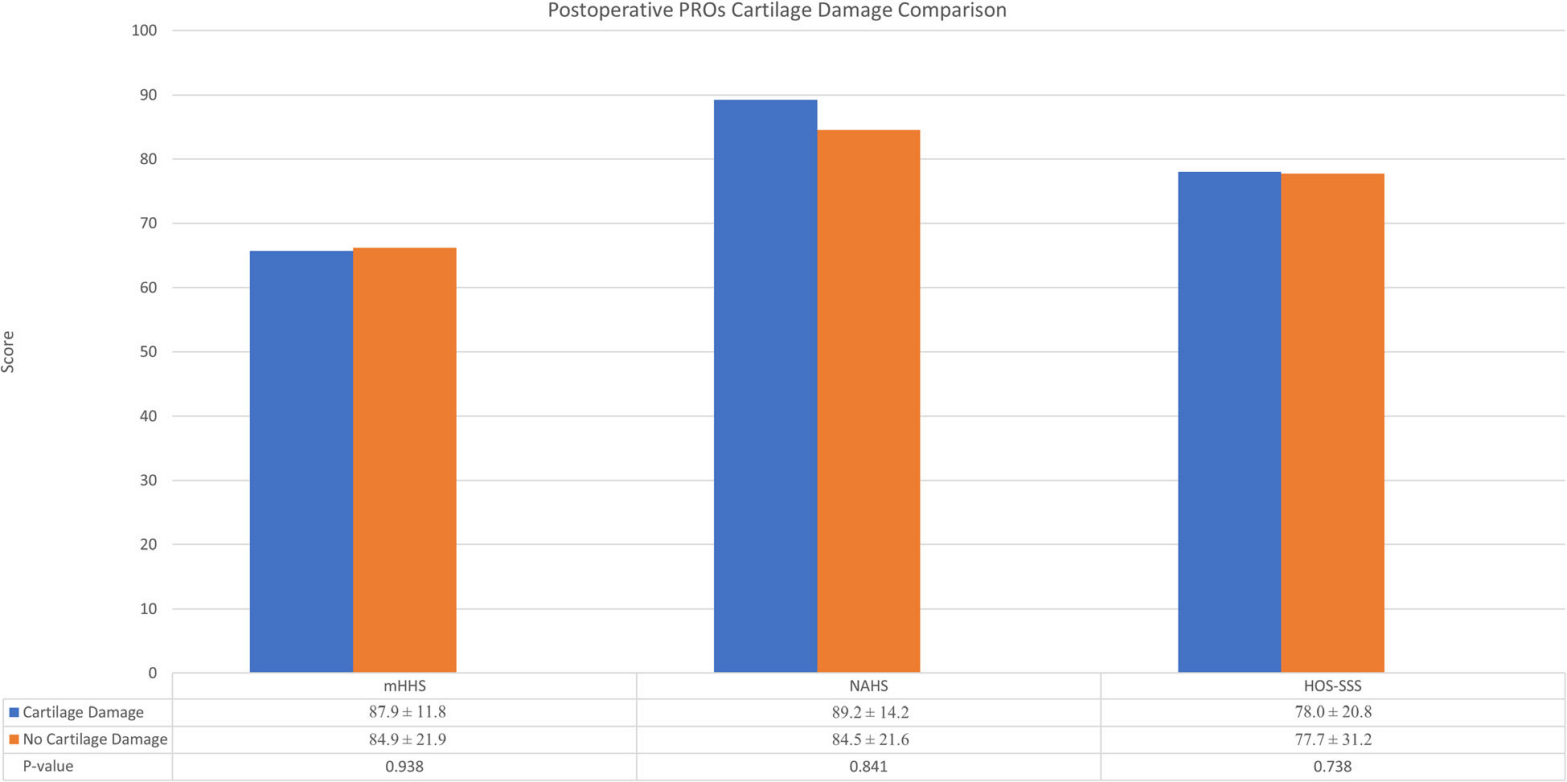
(PROs), patient reported outcomes,, (mHHS), modified Harris Hip Score, (NAHS), Non-Arthritic Hip Score, (HOS-SSS), Hip Outcome Score-Sport Specific Subscale

The intraoperative findings suggested 17 cases had an acetabular Outerbridge or femoral head Outerbridge grade > 2 (11 acetabular Outerbridge > 2, 2 femoral head Outerbridge > 2, and 4 with both grades > 2). Demographic factors including age at surgery and BMI were not statistically different between the hips with severe cartilage damage and hips without severe cartilage damage (*P* = 0.112 and 0.052, respectively). Similarly, both groups returned to tennis at similar rates (*P* = 0.933). The group with severe cartilage damage returned to tennis at a 76.4% rate and cases without severe cartilage damage returned at a 72.7% rate. Pre- and postoperative PROs were compared between the two groups. No statistical differences were found between mHHS, NAHS, or HOS-SSS preoperatively or postoperatively as shown in Figs. 3 and 4 (*P* > 0.05).

Of the 17 cases with severe cartilage damage, 8 (47.1%) required follow-up treatment including 1 revision arthroscopy, 2 THA, 4 platelet-rich plasma injections, and 1 lidocaine and depo-Medrol injection to the piriformis. The need for a secondary surgery was similar between groups (*P* > 0.999).

### Complications, secondary arthroscopies, and conversions to total hip arthroplasty

Of the entire cohort, one (3.6%) patient had follow-up complications. This patient reported piriformis syndrome and bursitis at 6-year follow-up. One (3.6%) hip required a secondary arthroscopy at 9.9 months and four (14.3%) hips converted to a THA at mean of 31.3 months following the index surgery.

## Discussion

This study showed that PROs significantly improved from pre-operative to latest follow-up: mHHS from 67.0 to 86.7 NAHS from 65.9 to 87.2, HOS-SSS from 50.0 to 77.9, and VAS from 5.4 to 1.8. As expected, tennis players who were able to RTS had higher mHHS, NAHS, and HOS-SSS scores at latest follow-up, although only significant for NAHS and HOS-SSS. There were no significant differences in pre- to post-operative changes in PROs between competitive and recreational athletes. However, HOS-SSS and VAS were statistically better at latest follow-up between competitive athletes and recreational athletes. Additionally, the sub-analysis comparing athletes with severe cartilage damage to those without severe cartilage damage showed no differences in RTS rate, preoperative PROs, or postoperative PROs.

Previous studies have investigated functional outcomes in an athletic population following hip arthroscopy. Nho et al. reported an improvement in mHHS of 66.8 to 88.5 [35], a delta similar to the present study. Furthermore, in a cohort of 53 athletic patients at average 2.4 year follow-up, Brunner et al. reported comparable postoperative scores for NAHS as found in this study (86.7 versus 87.2) [36]. These results extend to professional athletes, as studies have shown high (> 80%) rates of return to play after hip arthroscopy. Philippon et al. reported a 93% RTS rate in 45 professional athletes [37]; Menge et al. reported a 85.7% RTS rate [38]; and Bokyin et al. reported a 85.7% RTS rate in patients who underwent hip arthroscopy with labral reconstruction [39]. Furthermore, literature has also reported similar RTS rates in athletic patients who underwent open surgery for FAI [40].

To our knowledge, there has not been a previous study to report PROs and RTS on tennis players who underwent hip arthroscopy. In the present study, there were 7 (25.0%) tennis players (1 high school, 1 organized amateur, and 5 recreational) who were not playing tennis at latest follow-up. Out of the 8 competitive tennis players, 7 (87.5%) were able to RTS. However, our cohort is small and is also likely affected by the small number of professional tennis players.

In the present study, the difference in return to play between competitive and recreational tennis players was not statistically significant (*P* = 0.233). However, this result must be interpreted cautiously due to the small number of professional athletes analyzed in this study. Professional athletes have been shown to RTS at higher rates than recreational athletes [35, 38]. Rate of RTS appears to be highly influenced by factors such as self-motivation, aging, pain, encouragement, and adaptation to physical limitations [41, 42]. These factors as well as monetary incentives and the limited window to play at the elite level motivate professional athletes to return to play. In a recent systematic review, Casartelli et al. reported a 87% RTS rate in both competitive and recreational athletes [10].

Tennis is considered a physically demanding sport, with more cutting and pivoting motions than biking or swimming [2]. Interestingly, tennis players with greater internal rotation and greater external rotation were less likely to RTS. Studies have evaluated the relationship between general joint hypermobility and athletic outcomes following hip arthroscopy. Weber et al. found that capsular plication in athletes may actually impair RTS by limiting the external rotation necessary for complex sports [43]. On the contrary, studies have also shown no significant relationship between Beightons scores and functional outcomes following hip arthroscopy [44, 45]. It seems that patients with greater internal or external rotation have increased joint instability that may preclude them from returning to their sport.

### Strengths

Strengths of this study include the use of multiple validated functional hip outcome scores such as mHHS, NAHS, and HOS-SSS, that were designed specifically to detect outcomes in active patients with non-arthritic hips. Moreover, with this multiple PROs use, the authors tried to limit the ceiling effect of a single PRO. Furthermore, this is one of the only studies to report PROs in a mixed group of tennis players at minimum five-year follow up. Finally, as statistical significance does not equate clinical significance, the proportion of patients who achieved the MCID and PASS for mHHS and HOS-SSS was also provided [34, 46].

### Limitations

Limitations include the non-randomized and retrospective design of the present study. Further, heterogeneity among the arthroscopic procedures performed and competitive level must be acknowledged and could influence the findings of the study. The study is also retrospective in nature, which introduces an inherent bias; nevertheless, this bias may be limited by prospective data collection. The present study included minimum five-year follow-up, albeit longer follow is needed to determine durability of the results. In addition, all procedures were performed by a single surgeon, which may limit the generalizability of the results. Although this is one of the few case-series reporting mid-term outcomes in tennis players who underwent hip arthroscopy, the sample size in relatively small and limits the generalizability of the results. Furthermore, capsular and labral management has evolved in the recent years and in consequence, patients who underwent labral debridement and capsular release in the ongoing study would currently be treated with labral restoration techniques such labral reconstruction or labral augmentation, and capsular plication [25, 26, 47–49].

## Conclusion

Regardless of the level of participation, tennis players who underwent hip arthroscopic surgery reported statistically significant PRO improvements. A favorable RTS was also achieved by players with a continued interest in playing. The data here may be useful in counseling tennis players of various levels who are considering arthroscopic treatment of a hip injury.

## Data Availability

No additional data is available.

## Abbreviations

PROs: Patient reported outcomes
FAI: Femoracetabular Impingement
RTS: Return to sport
mHHS: Modified Harris Hip Score
NAHS: Non-arthritic Hip Score
HOS-SSS: Hip Outcome Score – Sport Specific Subscale
VAS: Visual analog scale
PASS: Patient Accepted Symptomatic State
MCID: Minimal clinically important difference
THA: Total hip arthroplasty
TIA: Tennis Industry Association
MRA: Magnetic Resonance Angiogram
NSAIDs: Non-steroidal anti-inflammatory drugs
BMI: Body Mass Index
ALAD: Acetabular labral articular disruption
LT: Ligamentum Teres
SD: Standard deviation
∆: Delta
iHOT-12: International Hip Outcome Tool
VR-12P: Physical component of the Veterans RAND 12-Item Health Survey
VR-12 M: Mental component of the Veterans RAND 12-Item Health Survey
SF-12P: Physical component of the Short Form 12
SF-12 M: Mental components of the Short Form 12
